# A confidence interval analysis of sampling effort, sequencing depth, and taxonomic resolution of fungal community ecology in the era of high-throughput sequencing

**DOI:** 10.1371/journal.pone.0189796

**Published:** 2017-12-18

**Authors:** Ryoko Oono

**Affiliations:** Department of Ecology, Evolution, and Marine Biology, University of California, Santa Barbara, CA, United States of America; INRA, FRANCE

## Abstract

High-throughput sequencing technology has helped microbial community ecologists explore ecological and evolutionary patterns at unprecedented scales. The benefits of a large sample size still typically outweigh that of greater sequencing depths per sample for accurate estimations of ecological inferences. However, excluding or not sequencing rare taxa may mislead the answers to the questions ‘how and why are communities different?’ This study evaluates the confidence intervals of ecological inferences from high-throughput sequencing data of foliar fungal endophytes as case studies through a range of sampling efforts, sequencing depths, and taxonomic resolutions to understand how technical and analytical practices may affect our interpretations. Increasing sampling size reliably decreased confidence intervals across multiple community comparisons. However, the effects of sequencing depths on confidence intervals depended on how rare taxa influenced the dissimilarity estimates among communities and did not significantly decrease confidence intervals for all community comparisons. A comparison of simulated communities under random drift suggests that sequencing depths are important in estimating dissimilarities between microbial communities under neutral selective processes. Confidence interval analyses reveal important biases as well as biological trends in microbial community studies that otherwise may be ignored when communities are only compared for statistically significant differences.

## Introduction

Microbiology has been revolutionized by high-throughput sequencing (HTS), allowing the investigation of ecological and evolutionary patterns at unprecedented broader and deeper scales, especially for the rare or cryptic microbial biosphere [1,2]. For example, until a few years ago, sampling efforts significantly limited ecological and evolutionary inferences for the hyperdiverse foliar fungal endophytes (FFE) because of the time and labor necessary for culturing, isolating, and genotyping individual fungi from plant tissues. High-throughput amplicon sequencing (e.g., ITS rDNA sequencing), has transformed the field by allowing more samples to be sequenced at deeper coverage per community with greater resolution of genetic variation within communities. Still, undersampling and lack of replication remain potential sources of bias for microbial community ecologists. Pilot studies are suggested to understand variability and species diversity of samples [3], but resources are often limiting, either in the field or in the lab, for a preliminary power analysis. Understanding how current practices may bias our inferences of HTS data will help design better experiments and fieldwork [4,5]. The goal of this study is to use real fungal ITS rDNA sequencing datasets and simulated microbial community datasets to better understand the interactive effects of key design variables, such as sampling efforts and sequencing depths, on the robustness of a range of effect sizes, from prominent to subtle community structures.

One of the most commonly debated questions for efficient HTS usage is sampling effort versus sequencing depth (e.g., [5–9]). The number of samples and sequencing coverage are not necessarily tradeoffs if multiple sequencing runs can be computationally combined for the same samples. Nevertheless, the cost of sampling (e.g., experimental set-up, travel, tissue storage, DNA extraction, etc.) tends to stay the same while the cost of sequencing has historically decreased. Many analytical studies conclude that large numbers of samples at shallow coverage (1,000–2,000 sequences per sample) are better than small numbers of samples at deep coverage to detect biologically relevant patterns (e.g., [10,11]). This is because beta diversity (differences among or between samples), unlike alpha diversity, tends to be relatively insensitive to sequencing depths [11]. Deeper sequencing, however, is necessary to reveal rare (low-abundance) taxa, whose importance in community ecology is debated [12]. Even when sequencing depths are high enough to reveal a rich rare biosphere, rare taxa are sometimes deliberately excluded from community analyses since they may represent sequencing errors, contaminations (both pre- and in-sequencing, see [13,14]), or their exclusion improves statistical precision [15]. Discarding rare sequences is also recommended due to their likely artefactual origins (e.g., [16,17]). The widespread practice to rarefy community samples to a common number of minimum sequences also effectively removes the most rare taxa from final analyses. Some studies even suggest shallow sequencing will improve correlations between community composition and environmental parameters [18]. However, low-abundance taxa may be ‘conditionally rare’ and reveal ecologically important species during disturbance or seed banks that make robust ecosystems [12,19–21] or are likely ‘real’ biological signals [17,22]. Furthermore, some evolutionary processes, such as drift or passive dispersal, may disproportionately affect the populations of rare taxa more than abundant taxa [23], which leads to subtle differences among communities that would not be inferred without deep sequencing.

In addition to sampling effort and sequencing depth, microbiologists are interested in understanding how taxonomic resolutions influence the robustness and effect sizes of community dissimilarities [24]. How taxonomic resolution influences the strength (i.e., effect size) of ecological patterns could give clues to the underlying genetic structure of key taxa and the evolutionary forces underlying community assembly (e.g., [25]). For example, if finer taxonomic groupings increased beta diversity and gave greater resolution to the population genetic structure across landscapes or habitats, this reflects rapid niche divergence among closely-related taxa. On the other hand, if coarser taxonomic groupings either increased or had little effect on community structure, this reflects niche conservatism [24].

The goals of this study were to explore the precision (confidence intervals) and effect sizes across three technical variables of HTS studies pertaining to microbial communities: 1) sampling effort, 2) sequencing depth, and 3) taxonomic resolution. Foliar fungal endophyte communities were used as case studies because studies using HTS data on FFE are still relatively rare compared to those for bacterial or mycorrhizal fungi and the species (i.e., sequence) diversity among potential sampling units (e.g., tissue mass, leaves, canopies, or host species range) is difficult to extrapolate from preliminary culturing studies. Foliar fungal endophytes are also not a monophyletic assemblage, but are a diverse group, spanning at least two phyla and more than six classes [26–28]. The number of species sequenced from a single individual depends highly on the methods for isolation and identification, and internal transcribed spacer (ITS) amplicon sequencing has revealed several dozen to over one hundred distinct operational taxonomic units (OTUs) per host individual or sample [29,30]. The sequence diversity also depends on a number of technical practices (e.g., primer discrimination between plant and fungal DNA, plant tissue size, PCR conditions, etc.) and is challenging to predict prior to sequencing. The unknown sequence diversity presents barriers to designing sampling strategies and performing power analyses. To better under the effects of technical variables, such as sequencing depths, on ecological or evolutionary interpretations from HTS community data, ITSrDNA datasets from Illumina MiSeq HTS were selected from two ongoing studies that vary in effect sizes: FFE communities with 1) low dissimilarity between different tissue types of the same host or 2) high dissimilarity between different geographic locations from the same host species.

The effect sizes (i.e., microbial community dissimilarity) between groups and their confidence intervals were non-parametrically analyzed using analysis of similarities (ANOSIM; [31]) and permutational ANOVA (PERMANOVA; [32]). The ANOSIM R value is based on the difference in the average ranking of dissimilarity indices between- and within-group communities. ANOSIM is appropriate for testing general differences between groups and not only differences between their centroids. PERMANOVA specifically tests if the centroids of the groups are equivalent for all groups and is known to be unaffected by heterogeneity in dispersions for balanced designs [33]. The effects of increasing sampling effort or sequencing depth, which increases the proportion of rare taxa in microbial communities, were tested with two dissimilarity indices that are commonly used: Bray-Curtis and Jaccard. A binary metric, such as Jaccard, that gives equal weight to rare and abundant taxa may increasingly overestimate community dissimilarity as sequencing depths increase compared to the Bray-Curtis index that gives more weight to abundant taxa. Increasing sequencing depths and sampling efforts were expected to both improve confidence intervals on estimates of community dissimilarity, although not necessarily linearly. The effects of taxonomic resolution were expected to vary by the types of communities that were compared. Confidence intervals (CI) rather than *p-*values were investigated to better understand the magnitude and precision of the differences in community structures [34]. Finally, the effects of sampling efforts and sequencing depths on effect sizes and CI were measured for simulated communities under random drift (i.e., no selection). The case studies here do not make recommendations on sampling size or sequencing depths, but demonstrate how these two factors along with taxonomic resolution can affect various ecological and evolutionary inferences from HTS data of microbial communities.

## Materials and methods

### Sampling and sequencing

The effects of sampling efforts, sequencing depths, and taxonomic resolution were tested on the precision of ecological inference using datasets from two Illumina MiSeq sequencing of foliar fungal endophytes (FFE). The FFE sequences came from tips and bases of *Pinus taeda* (loblolly pine) needles (n = 127) growing in North Carolina, USA, or whole *Pinus torreyana* (Torrey pine) needles (n = 38) from California, USA (Santa Rosa Island and San Diego). Sampling, sequencing, data filtering procedures can be found in [23] for *P*. *taeda* needles and were similar to sampling procedure for *P*. *torreyana* needles. *P*. *torreyana* needles were collected in summer of 2015 from Santa Rosa Island (34.00 N, 120.05 W; permit issued by National Park Service) or Torrey Pine State Reserve (32.92 N, 117.25 W; permit issued by Department of Parks and Recreation) in California, US. The *P*. *torreyana* stands were mature, at least 6 inches in diameter at breast height. From each Torrey pine tree, 10 needles (10 different fascicles) from different branches were collected haphazardly between 2 and 10 m from the ground using a pole saw. In total, leaves from 24 tree samples from Santa Rosa Island and 14 tree samples from San Diego were processed for FFE sequencing.

DNA from needles were extracted and prepared in the same way as in [23]. The ITS1 region was amplified using the ITS1F-KYO1 and ITS2-KYO1 [35] primers modified with Illumina overhang adaptors. The first stage of amplification was carried out in a total volume of 25 μl using 10 ng of DNA, 0.1 μM of BSA, 2.5 μl of 10x PCR buffer containing 15 mM MgCl_2_, 200 μM of each dNTP, 0.75 units of Choice-Taq DNA polymerase (Denville Scientific Inc, Holliston, MA, USA), and 0.5 μM of each primer. The PCR conditions were 3 min at 95°C, followed by 35 cycles of 30 s at 95°C, 30 s at 47°C, 30 s at 72°C, and a final elongation of 5 min at 72°C. Non-template PCR reactions did not amplify any bands. Amplicons from duplicate parallel PCR reactions per sample were pooled and 5 μl were used as template in the second amplification, which consisted of 3 min at 95°C, followed by 10 cycles of 30 s at 95°C, 30 s at 55°C, 30 s at 72°C, and a final elongation for 5 min at 72°C. Amplicons were purified with Agencourt AMPure XP SPRI magnetic beads (Beckman Coulter, Brea, CA, USA) using a 1:1 ratio, and normalized to 4 nM. Paired-end sequencing (2 × 250 bps) was carried out on an Illumina MiSeq sequencer at the UC San Diego Sequencing Facility.

The sequencing analysis protocol is modified from the UPARSE pipeline recommended by Edgar [36] and implemented in USEARCH v9.0.2132 ([37]; http://drive5.com/uparse). Paired-end raw reads were assembled using *fastq_mergepairs*. Simultaneously, assembled reads shorter than 50 bps and having either more than 200 bps or 30% difference within the overlapping region were excluded. Because the overlapping regions averaged about 150 bps, most paired reads were excluded if there were more than 30% difference (45 bps). Merged reads were filtered for reads having a maximum expected error score lower than 1.0 [38]. Reads were clustered at 100% similarity using the *unoise* command with a minimum abundance size of 2 (default is 8), which also detects and discards chimeras based on a chimeric model built from the most abundant reads. Reads were then clustered together in molecular operational taxonomic units (OTUs) with various similarity cutoffs of 90%, 95%, 97% or 99% using the *cluster_simem* command, as recommended by the UPARSE manual. The centroid sequence of each cluster (i.e., the most common sequence from each OTU picked by the cluster_otus command) was used in a BLAST [39] search against the entire GenBank nucleotide database excluding sequences that originated from environmental sampling (ftp://ftp.ncbi.nlm.nih.gov/blast/db/nt*, downloaded on January 8, 2016) and outputs were parsed in MEGAN 4 [40] for taxonomic assignment (minimum score threshold of 170, minimum hit support of 1 read, max percent of best score 5%). Fungal reads were exported to construct an OTU abundance table using *usearch_global* with unfiltered reads.

See S1–S3 Tables for number of OTUs and reads in each sample. The datasets were chosen based on varying effect sizes of community dissimilarity. The FFE communities between *P*. *torreyana* stands in San Diego and Santa Rosa Island show high dissimilarity (ANOSIM R > 0.70, PERMANOVA R^2^ > 0.17) whereas the FFE communities between the tips and bases of *P*. *taeda* trees had statistically significant but relatively lower dissimilarity (ANOSIM R < 0.4, PERMANOVA R^2^ < 0.07). Both datasets were compared with known samples containing a single ITS clone in their respective sequencing libraries to assess potential mistagging (e.g., [14]) and error rates.

### Statistical analysis

To test the effect of sampling effort on statistical confidence, I analyzed the ANOSIM R and PERMANOVA R^2^ values for 1000 random subsamples of each group (e.g., tip *vs*. base of *P*. *taeda* needles or *P*. *torreyana* needles from Santa Rosa *vs*. San Diego) with replacement, varying in sampling size. The reads per sample were normalized with cumulative sum scaling rather than rarefying [41]. To test the effect of sequencing depth on statistical confidence, each sample was rarefied 1000 times to varying sequencing depths (i.e., 100, 1000, 5000, 10000, and 20000) and then subsampled to show their confounding effects at varying sampling efforts. The 95% confidence intervals were calculated from the 1000 random subsamples. Code for analyses were written in R software package and used the R packages metagenomeSeq [41] and vegan [42]. R codes can be found at DataONE Dash, Dataset, https://doi.org/10.15146/R3Z96M.

### Simulated community

To demonstrate how deep sequencing could be important in understanding effects of stochastic processes, as opposed to selection, drifted communities were simulated by randomly resampling from a community of 954 taxa and five million individuals. The OTU table from the *P*. *taeda* study at 97% ITS2 rDNA similarity was chosen because it had close to 1000 taxa. To simulate drift, five million individuals were randomly sampled with replacement for 20 generations to create two drifted communities from the same original community. The drifted communities were compared from 5, 10, 15 or 20 subsamples. The community under drift has no new species either from diversification or migration from outside communities. Naturally, the numbers of taxa and alpha diversities for the drifted communities are lower than the original community, but are comparable between the two drifted communities. Their beta diversities were compared with Jaccard and Bray-Curtis indices as in previous analyses but without normalizing or rarefying.

## Results and discussion

### Sampling effort

**Fig 1:**
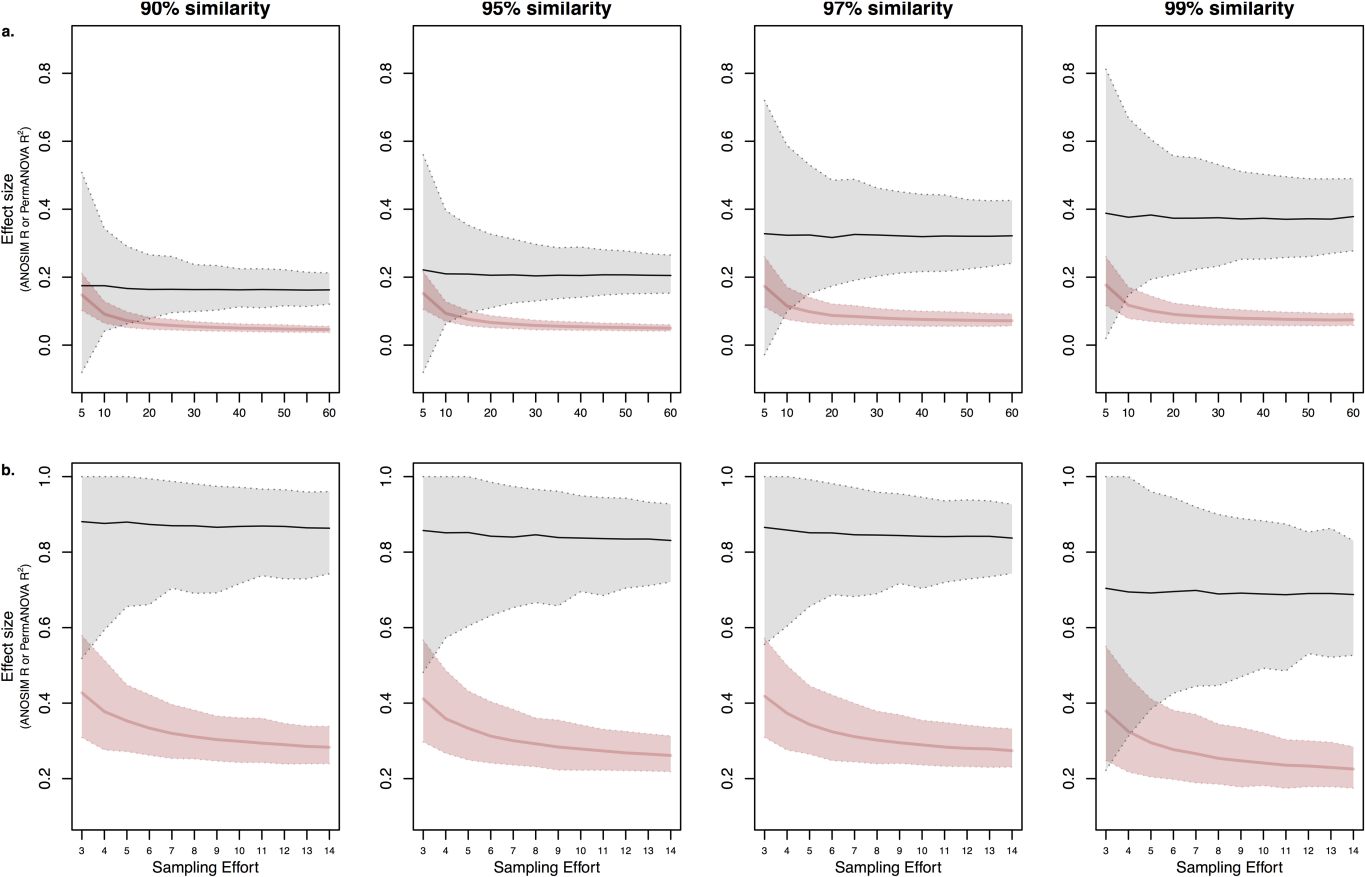
Effect of sampling effort on estimates of ANOSIM R and PERMANOVA R^2^ effect sizes and 95% confidence intervals. Sampling effort represents random subsamples of each comparison group with replacement. ANOSIM R (grey) and PERMANOVA R^2^ (red) values were calculated with Bray-Curtis dissimilarity and 95% confidence interval based on 1000 subsamples. a) Comparison of FFE communities between bases and tips of *P*. *taeda* needles at five-sample intervals from five to 60 samples each. b) Comparison of FFE communities between *P*. *torreyana* needles between different geographic locations (Santa Rosa and San Diego, CA) at one-sample intervals from three to 14 samples each.

As expected, increased sampling efforts significantly improved the precision of effect size estimations (Fig 1). Small sample sizes (3–5) produced imprecise and misleading estimates of effect sizes even when communities were highly differentiated (Fig 1B). The confidence interval (CI) widths plateaued for large sample sizes (>15) for both ANOSIM and PERMANOVA estimates (S1 Fig). Although the ANOSIM estimates were imprecise at low sampling sizes and improved considerably with increasing sampling efforts, the CI for PERMANOVA estimates were relatively precise even at low sampling sizes. The CI of PERMANOVA estimates also tended to be much more stable with increasing sampling effort than ANOSIM estimates, likely due to PERMANOVA being a specific test of differences in centroids among groups and less sensitive to heterogeneity of multivariate dispersions. The CI showed similar trends over increasing sampling efforts for community dissimilarities based on Bray-Curtis (Fig 1) and Jaccard (S2 Fig). The rate at which precision improved with increasing sampling efforts most likely had much to do with the differential abundance of key OTUs among samples and their abundance relative to other OTUs that increase background noise.

### Sequencing depth

**Fig 2:**
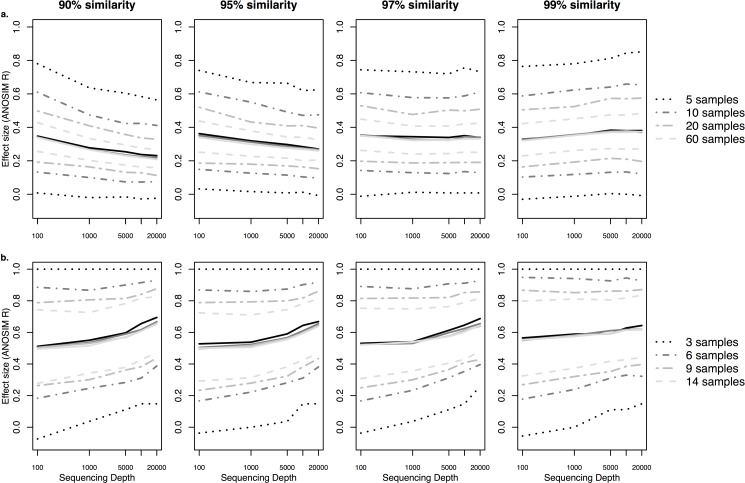
Effect of sequencing depths on estimates of ANOSIM R and 95% confidence intervals based on Bray-Curtis dissimilarity indices. Comparisons were made between FFE communities of a) bases and tips of *P*. *taeda* needles at 5, 10, 20 and 60 samples per group and b) between *P*. *torreyana* needles between Santa Rosa and San Diego, CA at 3, 6, 9, and 14 samples per group. Sequencing depths were tested at 100, 1000, 5000, 10000, 20000. X-axes are on a log-scale. Dotted lines indicate 95% confidence intervals for different sampling sizes. Solid lines indicate the mean. Trends for different sampling efforts were overlapped in a single plot.

The sequencing depths (i.e., randomly rarefying without replacement the final OTU table to a common number of sequences per sample) had less effect on CI than did sampling efforts (Fig 2, S3 and S4 Figs). However, estimates of effect sizes became more precise overall with increasing sequencing depths, especially for communities at coarser taxonomic resolution (90%). Sometimes precision decreased at high sequencing depths for OTUs at finer taxonomic resolution (99%; Fig 2A, S3B and S3D Fig), which was likely due to increasing noise by random or artefactual OTUs. Increased sequencing depths improved the precision for estimating the difference between FFE communities of *P*. *torreyana* trees at different geographic regions (Fig 2B) more than for tips *vs*. bases of needles on the same trees of *P*. *taeda* (Fig 2A).

Changes in CI are influenced by several factors that change with sequencing depths, namely the relative number and abundance of influential taxa in the community and random noise that diminishes precision of estimations with additional rare taxa. When effect sizes are estimated with ANOSIM, narrowing CI with increasing sequencing depths suggests that rare taxa improve the estimation of average rankings (i.e., narrowing the sampling distribution of Jaccard or Bray-Curtis indices) of within- and between-group dissimilarities. On the other hand, when effect sizes are estimated with PERMANOVA, narrowing CI with increasing sequencing depths suggests that rare taxa stabilizes the dissimilarity metrics between and within-groups.

The CI between Jaccard and Bray-Curtis analyses were considerably more similar across sampling efforts (S1 Fig) than across sequencing depths (S3 and S4 Figs), where Jaccard estimations tended to be less variable than Bray-Curtis (S3 and S4 Figs). This revealed that estimation of average rankings (ANOSIM) or partitioned variations (PERMANOVA) of community dissimilarity based on OTU presence/absence are not as variable as based on relative abundances. It is logical to assume that precision is influenced more by the dissimilarity metric when there are fewer rare taxa (low sequencing depths) than when rare taxa are observed more often (high sequencing depths).

Many analytical studies report ‘moderate’ sequencing (e.g., 1,000–2,000 reads per sample) is sufficient to give similar inferences [10,11] to deeper sequencing data. When microbial species are specialized to certain habitats, their abundance is expected to increase due to optimized performances. On the other hand, generalist microbial species are found in diverse environments but at lower abundances in any one habitat. Hence, shallow sequencing still has a very high likelihood of uncovering differences in abundance or presence of specialist taxa to capture similar effect sizes among communities as deep sequencing [43]. However, rare taxa sometimes can have large influences on community dissimilarities, such as communities across geographic regions [44], where drift and dispersal are the key factors differentiating microbial communities. Sequencing depths may have had a greater effect on narrowing CI widths for FFE communities between Santa Rosa and San Diego (Fig 2B) than communities between tip and base sections of needles on the same trees (Fig 2A) because of the influence of rare taxa in the former community comparison.

**Fig 3:**
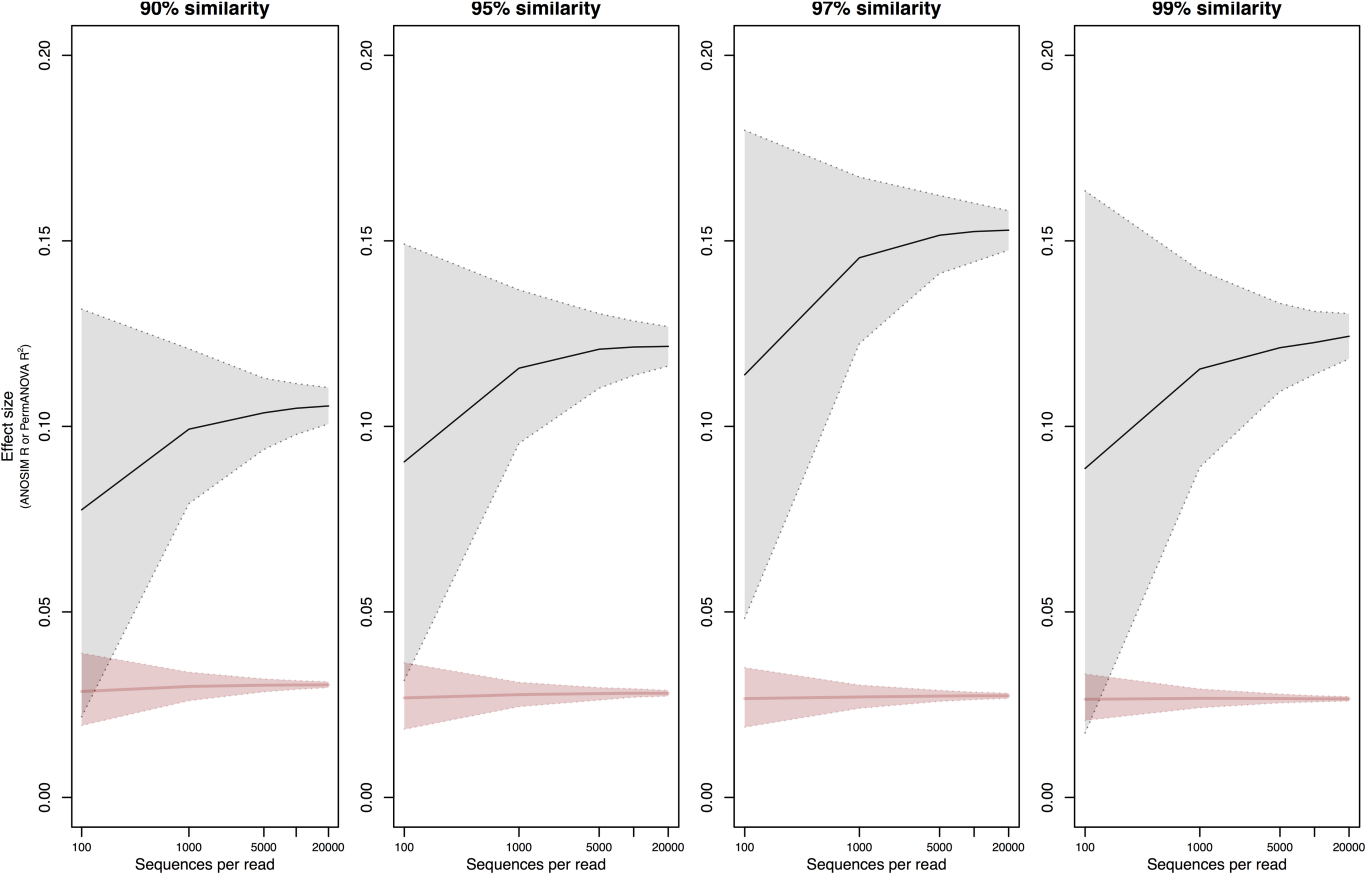
Effect of sequencing depths on estimates of geographic community dissimilarity and 95% confidence intervals for FFE communities that differ in geography. FFE communities between nine *P*. *taeda* plots across varying distances (1–107 km) were compared. ANOSIM R (grey) and PermANOVA R^2^ (red) values were calculated with Bray-Curtis dissimilarity and 95% confidence interval based on 1000 subsamples. Sequencing depth tested at 100, 1000, 5000, 10000, 20000. X-axes are on a log-scale. Dotted lines indicate 95% confidence interval. Solid lines indicate the mean.

To further test if rare taxa could influence effect sizes of communities across geographic regions, we compared the beta diversity of FFE communities among nine *P*. *taeda* plots located at various distances from one another (same dataset from [23]). The effect of sampling effort on the nine plots was not tested since there were only four trees per plot. Interestingly, unlike the previous community comparisons, increasing sequencing depths markedly improved the precision of dissimilarity estimates among FFE communities (Fig 3). The CI decreased more with sequencing depths when ANOSIM R or PERMANOVA R^2^ was estimated with Bray-Curtis (average CI decrease of 91% from 100 to 20,000 sequences) than with Jaccard (average CI decrease of 46% from 100 to 20,000 sequences; S5 Fig). This further supports the importance of rare taxa in such community comparisons since Jaccard gives more weight to rare taxa than Bray-Curtis.

### Taxonomic resolution

The CI tended to widen at finer taxonomic resolutions (99%; Figs 1–3, S1 and S2 Figs), which is likely due to increasing noise by random or artefactual OTUs with finer clustering algorithms and chimera filtering. This could be due to increased number of unique OTUs in each of the groups due to splitting of taxonomic groups or greater niche divergence among influential taxa. Precision based on PERMANOVA analyses (S6 Fig) tended to be less affected by taxonomic resolution than ANOSIM analyses (S7 Fig), suggesting that finer taxonomic resolution (99% cut-offs) increase heteroscedasticity among samples (i.e., noise) rather than affect the overall centroid locations of each sample. This further echos recommendations by others to be vigilant during clustering and filtering [45,46].

### Simulation of drift

**Fig 4:**
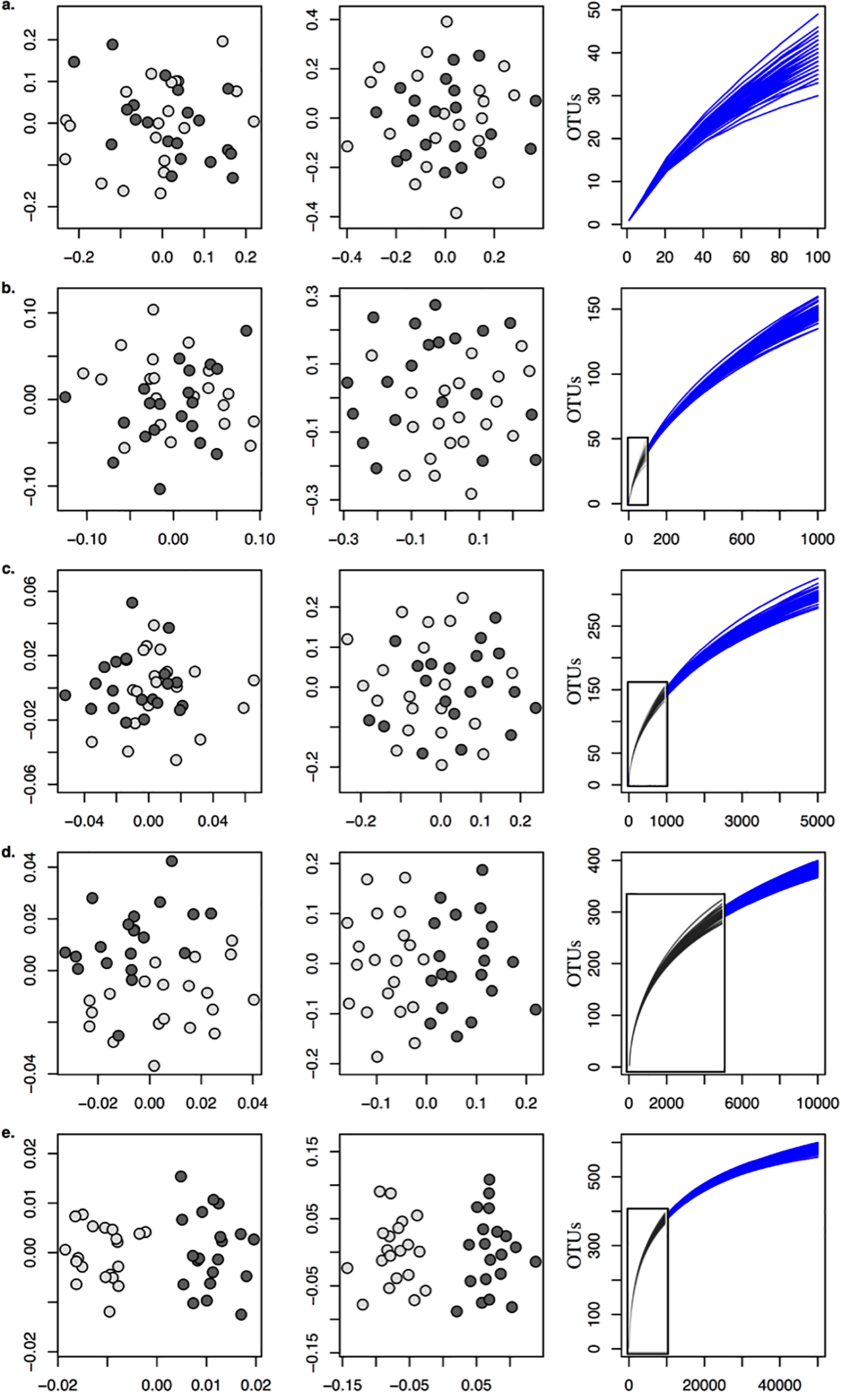
β-diversity patterns are revealed with increasing sequencing depths between communities that differ only by random processes. Non-metric multidimensional scaling of two simulated communities drifted for 20 generations from one community with 5 million individuals and 974 OTUs (taxa) have beta-diversity patterns revealed only after 50,000 sequences per sample, which corresponds to ~1% of the community per sample. Ordinations are based on between-sample dissimilarity calculated with Bray-Curtis (left panel), Jaccard (middle panel). OTU accumulation curves (right panel) demonstrate community sampling with varying sequencing depths. From top to bottom, sequencing depths correspond to a) 100, b) 1000, c) 5000, d) 10000, and e) 50000 sequences. Insets represent the accumulation curves from the previous sequencing depth.

To test how sequencing depth affects the estimation and robustness of the effect size of random processes, such as genetic drift, two communities were simulated to drift for 20 generations from a common community and subsampled at varying sequencing depths. As expected, drifted communities were only distinguishable with deep sequencing (> 10,000 sequences per sample; Fig 4) when at least 1% of individuals were sampled from the simulated community. The Jaccard dissimilarity distinguished between drifted communities slightly faster than Bray-Curtis due to the greater weight on the rare taxa.

The effect of sequencing depths on CI, however, was not immediately apparent (S8 Fig) since the addition of rare taxa maintained the variance of the sampling distribution of the relative rankings of within- and between-group dissimilarities (S9 Fig). The CI did not narrow until more than 10,000 individuals were sequenced per sample.

## Conclusions

In this study, confidence intervals and precision were emphasized over hypothesis-testing and *p* values to understand the nature of community dissimilarities estimated with large sampling sizes or deep sequencing depths in the era of HTS. While p-values are robust to testing the probability of differences from null predictions, confidence intervals inform the strength of this effect, which can be compared between different treatments. For example, one might compare the strength of effect size between different environmental factors by looking at how 95% CI values overlap with subsampling.

The results emphasize the importance of sampling efforts on precision, especially from low to mid-size sampling efforts. The effect of taxonomic resolution and sequencing depth on CI may also give clues to patterns of niche conservatism or divergence among taxa, the relative abundance and number of influential taxa on community dissimilarities, as well as the efficacy of filtering and clustering methods. This study also reveals how sequencing depths can bias our inferences. Studies that explore selection and adaptation (specialization) may not require deep sequencing because specialist taxa tend to be abundant [43] although low-abundant (rare) taxa can also be host-specific [1,12]. Conclusions from culture-dependent studies with lower sampling per community are still likely relevant and significant. In contrast, studies that explore random processes influencing community assembly, such as drift or passive dispersal (e.g., geographic barriers and local species extinctions), require deeper sequencing. This is because abundant taxa are less likely to go to extinction by random processes compared to rare taxa in a given period. These statistical effects are likely observed in other diverse microbial communities characterized by few abundant taxa and many rare taxa and are not unique to foliar fungal endophytes.

A carefully randomized multiplex library should prevent low-abundance taxa with artificial origins, such as mistagging [13,14], contaminations, or sequencing errors, from having spurious correlations that would lead to biased ecological inferences. Low-abundance taxa may diminish the statistical power to detect key differences between samples (i.e., type II errors), but a large enough sample size should overcome this as well as help avoid type I errors. On the other hand, sequencing coverage alone cannot overcome potential spurious conclusions due to mistagging or contamination, no matter how deep. In moving forward, microbial community ecologists need to carefully prepare HTS libraries by the inclusion of mock or positive-control communities, sample replicates, and balanced primer usage frequency to detect ambiguous rare OTUs and maximize the sequence information for analyses [14]. A prudent consideration of the filtering and clustering practices will allow for the inclusion of rare taxa in their final analyses for unbiased estimations of community dissimilarities.

## Supporting information

S1 Fig Effect of sampling effort on 95% confidence intervals of ANOSIM R and PermANOVA R^2^ estimates differentiating FFE communities between a) bases and tips of *P*. *taeda* needles and b) *P*. *torreyana* needles from San Diego and Santa Rosa Island. The dissimilarities were calculated using Bray-Curtis (a & c) or Jaccard (b & d). The CI depends on taxonomic resolution (different colored line) and the statistical test (different panels).
(PDF)

S2 Fig Effect of sampling effort on estimates of ANOSIM R and PermANOVA R^2^ effect sizes and 95% confidence intervals. Sampling effort represents random subsamples of each comparison group with replacement. ANOSIM R (grey) and PermANOVA R^2^ (red) values were calculated with Jaccard dissimilarity and 95% confidence interval based on 1000 subsamples. Jaccard indices were calculated with CSS normalized OTU tables. a) Comparison of FFE communities between bases and tips of *P*. *taeda* needles at five-sample intervals from five to 60 samples each. b) Comparison of FFE communities between *P*. *torreyana* needles between different geographic locations (Santa Rosa and San Diego, CA) at one-sample intervals from three to 14 samples each.
(PDF)

S3 Fig Effect of sequencing depth on 95% confidence intervals of ANOSIM R (a & c) and PermANOVA R^2^ (b & d) estimates differentiating FFE communities between bases and tips of *P*. *taeda* needles. The dissimilarities were calculated using Bray-Curtis (a & b) or Jaccard (c & d). The CI depends on sequencing depths (x-axis on log-scale), sampling effort (different markers), and taxonomic resolution (different panels).
(PDF)

S4 Fig Effect of sequencing depth on 95% confidence intervals of ANOSIM R (a & c) and PermANOVA R^2^ (b & d) estimates differentiating FFE communities between *P*. *torreyana* needles from Santa Rosa Island and San Diego, CA. The dissimilarities were calculated using Bray-Curtis (a & b) or Jaccard (c & d). The CI depends on sequencing depths (x-axis on log-scale), sampling effort (different markers), and taxonomic resolution (different panels).
(PDF)

S5 Fig Effect of sequencing depths on estimates of geographic community dissimilarity and 95% confidence intervals for FFE communities that differ in geography. FFE communities between nine *P*. *taeda* plots across varying distances (1–107 km) were compared. ANOSIM R (grey) and PermANOVA R^2^ (red) values were calculated with Jaccard dissimilarity and 95% confidence interval based on 1000 subsamples. Sequencing depth tested at 100, 1000, 5000, 10000, 20000. X-axes are on a log-scale. Dotted lines indicate 95% confidence interval. Solid lines indicate the mean.
(PDF)

S6 Fig Effect of taxonomic resolution on the 95% confidence interval of PERMANOVA R^2^ estimates based on Bray-Curtis (a & c) or Jaccard (b & d) dissimilarity between FFE communities; bases *vs*. tips of *P*. *taeda* needles (a & b) or *P*. *torreyana* needles from San Diego *vs*. Santa Rosa Island (c & d). The CI depends on sampling effort (different panels), sequencing depths (different markers), and taxonomic resolution (x-axis).
(PDF)

S7 Fig Effect of taxonomic resolution on the 95% confidence interval of ANOSIM R estimates based on Bray-Curtis (a & c) or Jaccard (b & d) dissimilarity for FFE communities; bases *vs*. tips of *P*. *taeda* needles (a & b) or *P*. *torreyana* needles from San Diego *vs*. Santa Rosa Island (c & d). The CI depends on sampling effort (different panels), sequencing depths (different markers), and taxonomic resolution (x-axis).
(PDF)

S8 Fig Effect of sequencing depth on estimates of ANOSIM R and its 95% confidence intervals for simulated communities under random drift for 20 generations using Bray-Curtis and Jaccard. Sequencing depth tested at 100, 1000, 5000, 10000, 50000, and 100000 sequences per sample. Dotted lines indicate 95% confidence intervals for different sampling sizes. Solid lines indicate the mean. Trends for different sample sizes were overlapped in a single plot.
(PDF)

S9 Fig Effect of sequencing depth on the Bray-Curtis dissimilarity values within and between two simulated communities under random drift for 20 generations. Sequencing depth tested at 100, 1000, 5000, 10000, and 50000 sequences per sample with 20 samples per community. R and p-values indicate the ANOSIM R and p-values with 1000 permutations. Density plots indicate the distribution of Bray-Curtis dissimilarity values within communities. Red density plot indicates Bray-Curtis dissimilarity values between the two communities.
(PDF)

S1 Table Number of ITS1/2 operational taxonomic units (OTUs) in entire dataset depending on similarity cut-off thresholds, and their effect sizes based on ANOSIM or PERMANOVA using Bray-Curtis dissimilarity. (PDF)

S2 Table Number of operational taxonomic units (S obs) and sequence reads per sample at different ITS2 similarity cut-offs for *P*. *taeda* needle section samples. Sample names are as follows: Plot, Tree replicate, Bottom or Top branch, Base or Tip of needles. For example, tissue sample 13TB represents needle sections from plot 1, tree 3, top canopy, and base of needles.
(PDF)

S3 Table Number of operational taxonomic units (S obs) and sequence reads per sample (read number) at different ITS1 similarity cut-off thresholds for *P*. *torreyana* needle samples. (PDF)
