# Supplementary material for: A confidence interval analysis of sampling effort, sequencing depth, and taxonomic resolution of fungal community ecology in the era of high-throughput sequencing
Source: PLoS One. 2017 Dec 18;12(12):e0189796. doi: 10.1371/journal.pone.0189796 (PMC5734782; doi:10.1371/journal.pone.0189796)
Supplement: S1 Fig — The dissimilarities were calculated using Bray-Curtis (a & c) or Jaccard (b & d). The CI depends on taxonomic resolution (different colored line) and the statistical test (different panels). (PDF) [file pone.0189796.s001.pdf]

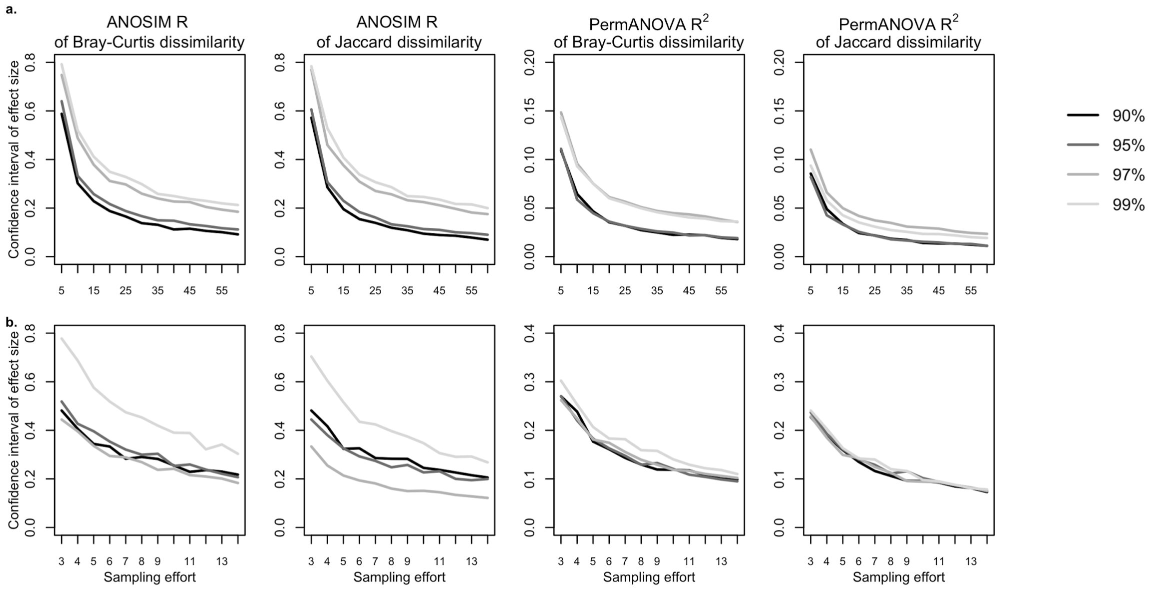

S1 Fig. Effect of sampling effort on 95% confidence intervals of ANOSIM R and PermANOVA R^2 estimates differentiating FFE communities between a) bases and tips of *P. taeda* needles and b) *P. torreyana* needles from San Diego and Santa Rosa Island. The dissimilarities were calculated using Bray-Curtis (a & c) or Jaccard (b & d). The CI depends on taxonomic resolution (different colored line) and the statistical test (different panels).
