# Supplementary material for: A confidence interval analysis of sampling effort, sequencing depth, and taxonomic resolution of fungal community ecology in the era of high-throughput sequencing
Source: PLoS One. 2017 Dec 18;12(12):e0189796. doi: 10.1371/journal.pone.0189796 (PMC5734782; doi:10.1371/journal.pone.0189796)
Supplement: S2 Fig — Sampling effort represents random subsamples of each comparison group with replacement. ANOSIM R (grey) and PermANOVA R2 (red) values were calculated with Jaccard dissimilarity and 95% confidence interval based on 1000 subsamples. Jaccard indices were calculated with CSS normalized OTU tables. a) Comparison of FFE communities between bases and tips of P. taeda needles at five-sample intervals from five to 60 samples each. b) Comparison of FFE communities between P. torreyana needles between different geographic locations (Santa Rosa and San Diego, CA) at one-sample intervals from three to 14 samples each. (PDF) [file pone.0189796.s002.pdf]

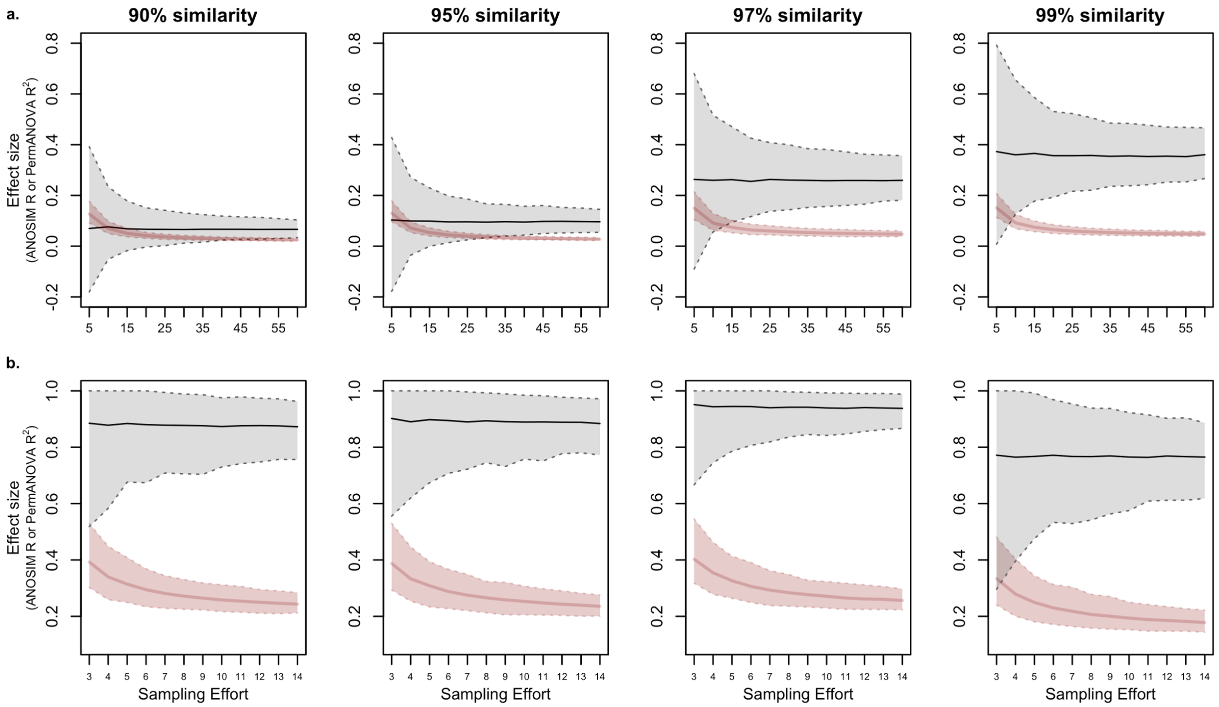

S2 Fig. Effect of sampling effort on estimates of ANOSIM R and PermANOVA R^2 effect sizes and 95% confidence intervals. Sampling effort represents random subsamples of each comparison group with replacement. ANOSIM R (grey) and PermANOVA R^2 (red) values were calculated with Jaccard dissimilarity and 95% confidence interval based on 1000 subsamples. Jaccard indices were calculated with CSS normalized OTU tables. a) Comparison of FFE communities between bases and tips of *P. taeda* needles at five-sample intervals from five to 60 samples each. b) Comparison of FFE communities between *P. torreyana* needles between different geographic locations (Santa Rosa and San Diego, CA) at one-sample intervals from three to 14 samples each.
