# Supplementary material for: A confidence interval analysis of sampling effort, sequencing depth, and taxonomic resolution of fungal community ecology in the era of high-throughput sequencing
Source: PLoS One. 2017 Dec 18;12(12):e0189796. doi: 10.1371/journal.pone.0189796 (PMC5734782; doi:10.1371/journal.pone.0189796)
Supplement: S3 Fig — The dissimilarities were calculated using Bray-Curtis (a & b) or Jaccard (c & d). The CI depends on sequencing depths (x-axis on log-scale), sampling effort (different markers), and taxonomic resolution (different panels). (PDF) [file pone.0189796.s003.pdf]

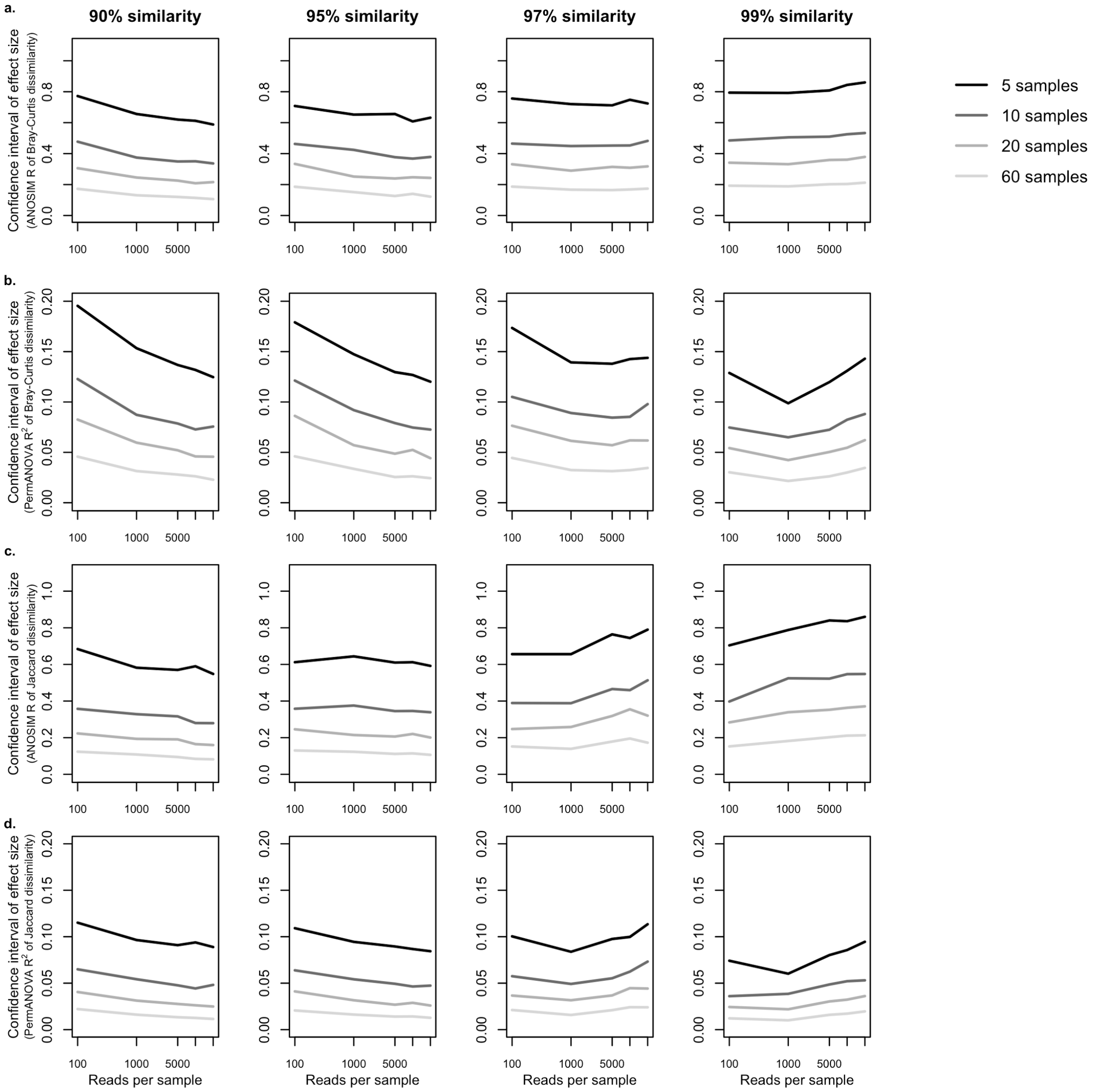

S3 Fig. Effect of sequencing depth on 95% confidence intervals of ANOSIM R (a & c) and PermANOVA R^2 (b & d) estimates differentiating FFE communities between bases and tips of *P. taeda* needles. The dissimilarities were calculated using Bray-Curtis (a & b) or Jaccard (c & d). The CI depends on sequencing depths (x-axis on log-scale), sampling effort (different markers), and taxonomic resolution (different panels).
