# Supplementary material for: A confidence interval analysis of sampling effort, sequencing depth, and taxonomic resolution of fungal community ecology in the era of high-throughput sequencing
Source: PLoS One. 2017 Dec 18;12(12):e0189796. doi: 10.1371/journal.pone.0189796 (PMC5734782; doi:10.1371/journal.pone.0189796)
Supplement: S5 Fig — FFE communities between nine P. taeda plots across varying distances (1–107 km) were compared. ANOSIM R (grey) and PermANOVA R2 (red) values were calculated with Jaccard dissimilarity and 95% confidence interval based on 1000 subsamples. Sequencing depth tested at 100, 1000, 5000, 10000, 20000. X-axes are on a log-scale. Dotted lines indicate 95% confidence interval. Solid lines indicate the mean. (PDF) [file pone.0189796.s005.pdf]

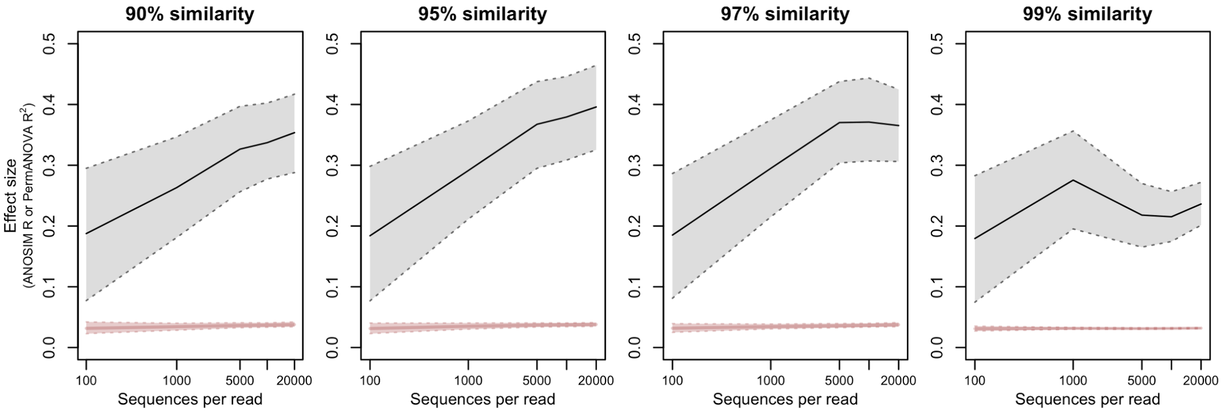

S5 Fig. Effect of sequencing depths on estimates of geographic community dissimilarity and 95% confidence intervals for FFE communities that differ in geography. FFE communities between nine *P. taeda* plots across varying distances (1 - 107 km) were compared. ANOSIM R (grey) and PermANOVA R^2 (red) values were calculated with Jaccard dissimilarity and 95% confidence interval based on 1000 subsamples. Sequencing depth tested at 100, 1000, 5000, 10000, 20000. X-axes are on a log-scale. Dotted lines indicate 95% confidence interval. Solid lines indicate the mean.
