# Supplementary material for: A confidence interval analysis of sampling effort, sequencing depth, and taxonomic resolution of fungal community ecology in the era of high-throughput sequencing
Source: PLoS One. 2017 Dec 18;12(12):e0189796. doi: 10.1371/journal.pone.0189796 (PMC5734782; doi:10.1371/journal.pone.0189796)
Supplement: S6 Fig — The CI depends on sampling effort (different panels), sequencing depths (different markers), and taxonomic resolution (x-axis). (PDF) [file pone.0189796.s006.pdf]

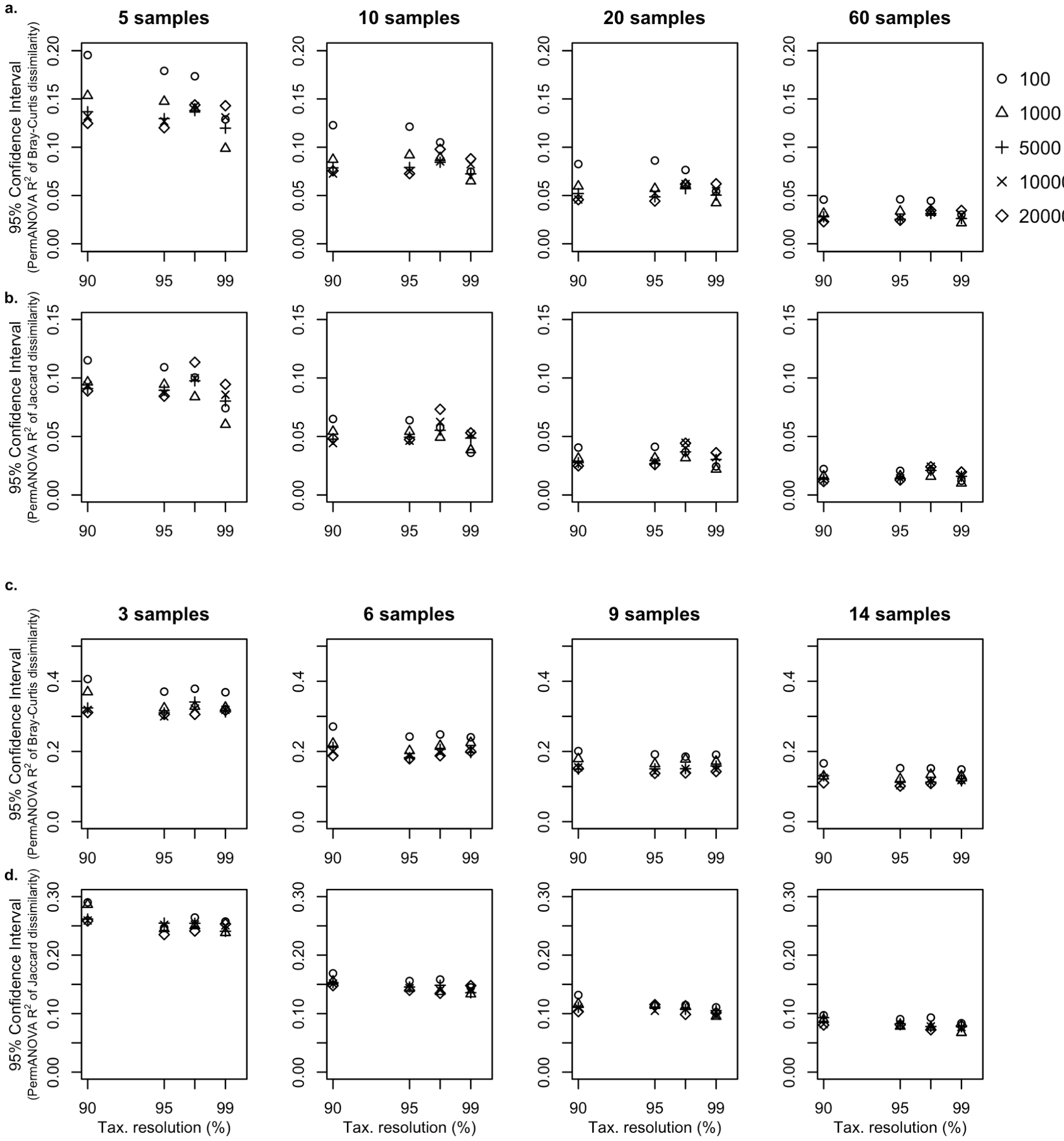

S6 Fig. Effect of taxonomic resolution on the 95% confidence interval of PERMANOVA R^2 estimates based on Bray-Curtis (a & c) or Jaccard (b & d) dissimilarity between FFE communities; bases *vs.* tips of *P. taeda* needles (a & b) or *P. torreyana* needles from San Diego *vs.* Santa Rosa Island (c & d). The CI depends on sampling effort (different panels), sequencing depths (different markers), and taxonomic resolution (x-axis).
