# Supplementary material for: A confidence interval analysis of sampling effort, sequencing depth, and taxonomic resolution of fungal community ecology in the era of high-throughput sequencing
Source: PLoS One. 2017 Dec 18;12(12):e0189796. doi: 10.1371/journal.pone.0189796 (PMC5734782; doi:10.1371/journal.pone.0189796)
Supplement: S8 Fig — Sequencing depth tested at 100, 1000, 5000, 10000, 50000, and 100000 sequences per sample. Dotted lines indicate 95% confidence intervals for different sampling sizes. Solid lines indicate the mean. Trends for different sample sizes were overlapped in a single plot. (PDF) [file pone.0189796.s008.pdf]

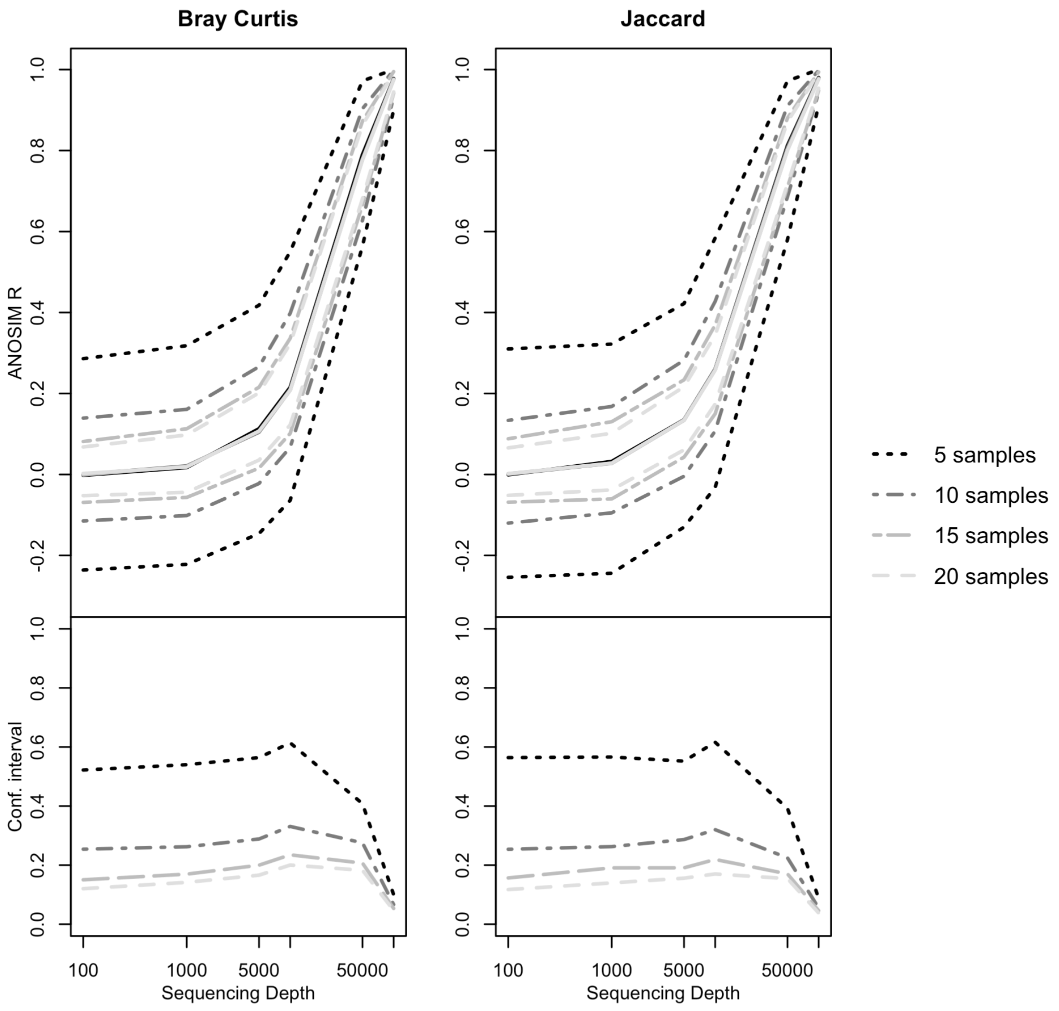

S8 Fig. Effect of sequencing depth on estimates of ANOSIM R and its 95% confidence intervals for simulated communities under random drift for 20 generations using Bray-Curtis and Jaccard. Sequencing depth tested at 100, 1000, 5000, 10000, 50000, and 100000 sequences per sample. Dotted lines indicate 95% confidence intervals for different sampling sizes. Solid lines indicate the mean. Trends for different sample sizes were overlapped in a single plot.
