# Supplementary material for: A confidence interval analysis of sampling effort, sequencing depth, and taxonomic resolution of fungal community ecology in the era of high-throughput sequencing
Source: PLoS One. 2017 Dec 18;12(12):e0189796. doi: 10.1371/journal.pone.0189796 (PMC5734782; doi:10.1371/journal.pone.0189796)
Supplement: S9 Fig — Sequencing depth tested at 100, 1000, 5000, 10000, and 50000 sequences per sample with 20 samples per community. R and p-values indicate the ANOSIM R and p-values with 1000 permutations. Density plots indicate the distribution of Bray-Curtis dissimilarity values within communities. Red density plot indicates Bray-Curtis dissimilarity values between the two communities. (PDF) [file pone.0189796.s009.pdf]

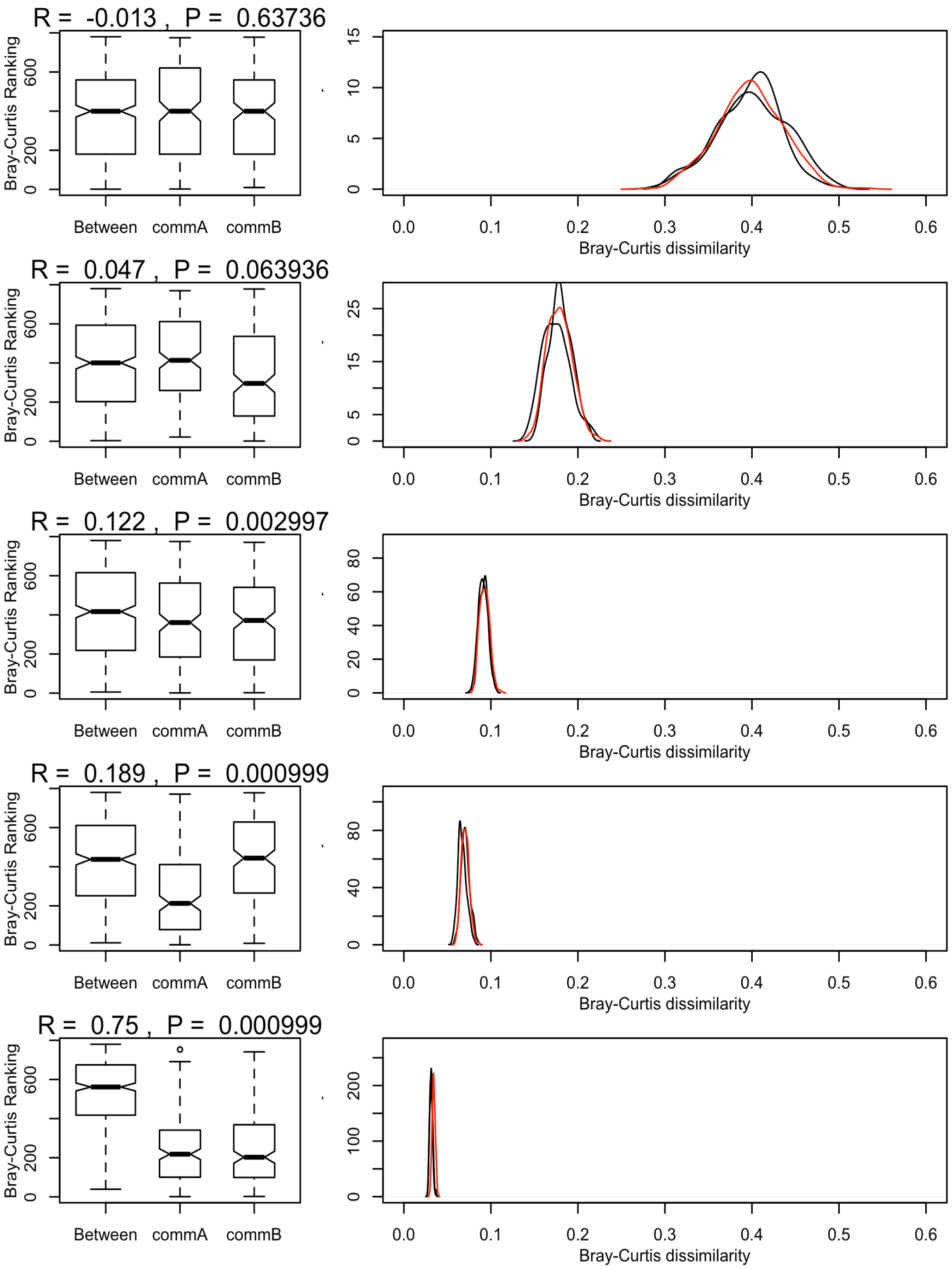

S9 Fig. Effect of sequencing depth on the Bray-Curtis dissimilarity values within and between two simulated communities under random drift for 20 generations. Sequencing depth tested at 100, 1000, 5000, 10000, and 50000 sequences per sample with 20 samples per community. R and P-values indicate the ANOSIM R and p-values with 1000 permutations. Density plots indicate the distribution of Bray-Curtis dissimilarity values within communities. Red density plot indicates Bray-Curtis dissimilarity values between the two communities.
