# Supplementary material for: A confidence interval analysis of sampling effort, sequencing depth, and taxonomic resolution of fungal community ecology in the era of high-throughput sequencing
Source: PLoS One. 2017 Dec 18;12(12):e0189796. doi: 10.1371/journal.pone.0189796 (PMC5734782; doi:10.1371/journal.pone.0189796)
Supplement: S1 Table — (PDF) [file pone.0189796.s010.pdf]

S1 Table. Number of ITS1/2 operational taxonomic units (OTUs) in entire dataset depending on similarity cut-off thresholds, and their effect sizes based on ANOSIM or PERMANOVA using Bray-Curtis dissimilarity.

Community data	ITS1 similarity cut-off	# of OTUs	ANOSIM R	p-value	PERMANOVA R^2	p-value
38 *P. torreyana* samples	90%	138	0.8221	0.000999	0.23204	0.0001
	95%	164	0.7952	0.000999	0.21278	0.0001
	97%	191	0.8242	0.000999	0.22621	0.0001
	99%	931	0.7039	0.000999	0.17921	0.0001
	ITS2 similarity cut-off					
127 *P. taeda* section samples	90%	471	0.1485	0.000999	0.03748	0.0001
	95%	613	0.1922	0.000999	0.04151	0.0001
	97%	954	0.3081	0.000999	0.06315	0.0001
	99%	24,742	0.3611	0.000999	0.06517	0.0001
