# Supplementary material for: A confidence interval analysis of sampling effort, sequencing depth, and taxonomic resolution of fungal community ecology in the era of high-throughput sequencing
Source: PLoS One. 2017 Dec 18;12(12):e0189796. doi: 10.1371/journal.pone.0189796 (PMC5734782; doi:10.1371/journal.pone.0189796)
Supplement: S2 Table — Sample names are as follows: Plot, Tree replicate, Bottom or Top branch, Base or Tip of needles. For example, tissue sample 13TB represents needle sections from plot 1, tree 3, top canopy, and base of needles. (PDF) [file pone.0189796.s011.pdf]

S2 Table. Number of operational taxonomic units (S obs) and sequence reads per sample at different ITS2 similarity cut-offs for *P. taeda* needle section samples. Samples names are as follows: Plot, Tree replicate, Bottom or Top branch, Base or Tip of needles. For example, tissue sample 13TB represents needle sections from plot 1, tree 3, top canopy, and base of needles.

	S obs 90%	read number	S obs 95%	read number	S obs 97%	read number	S obs 99%	read number
94BT	38	47931	41	47910	75	47707	727	44094
94BB	59	25796	60	25465	106	25544	540	24246
94TB	29	28493	34	28413	82	28515	848	27023
94TT	55	41507	56	41562	99	42377	948	41436
93BB	59	21766	70	21668	90	21739	561	20953
93BT	62	67556	63	65138	116	65117	1128	63420
93TB	69	33174	78	33192	113	33190	565	32173
93TT	35	58102	40	58094	96	58015	1035	54521
92BB	51	105726	60	104867	107	106518	1309	104036
92BT	41	28939	40	28665	75	28607	853	25901
92TB	53	46539	61	46277	91	46427	1053	44200
92TT	52	15371	57	15367	90	15384	433	14652
91BB	26	21886	27	21461	48	21900	673	20943
91BT	49	44503	54	44498	80	45191	746	43459
91TB	50	66711	57	69564	91	69213	1388	66163
91TT	41	37449	43	37282	92	37534	628	35818
84BB	46	41963	57	42291	92	47087	1141	45769
84BT	40	37014	46	36431	97	37391	1245	36384
84TB	54	55810	76	56056	111	56141	1481	53253
84TT	57	65466	68	65544	151	65487	1781	62332
83BB	47	38915	53	39344	106	39478	1378	38241
83BT	55	59651	58	59786	121	60028	1349	57789
83TB	48	43031	59	43275	117	43309	1457	41140
83TT	53	47090	55	47054	109	47017	1245	44217
82BB	50	64463	57	65341	130	65412	1566	64180
82BT	59	55387	65	55295	128	55350	982	52108
82TB	74	53968	88	53629	159	54787	1640	53124
82TT	62	34318	72	34310	138	34288	1097	32206

81BB	52	42551	63	43564	103	44908	1294	44030
81BT	69	85678	77	85544	141	85650	1233	80556
81TB	52	66767	64	67261	102	67872	1586	65148
81TT	59	43102	69	42863	119	43029	1165	44489
74BB	49	44153	58	43813	108	44905	1018	44190
74BT	71	46704	87	46410	149	47743	1258	43436
74TB	46	57117	61	57272	105	57416	1140	55701
74TT	51	74497	60	74176	142	73861	1386	69092
73BB	60	47060	71	48178	108	49302	1052	47163
73BT	61	96378	65	96293	123	96045	1680	89412
73TB	55	59309	69	61200	113	61810	1631	59311
73TT	47	48005	55	51864	111	51627	1451	50155
72BB	46	55360	50	55331	82	56286	1129	53842
72BT	46	39522	47	39496	100	39518	1014	37492
72TB	44	46179	53	45416	87	47083	939	46504
72TT	69	59800	80	60224	143	60667	1454	58354
71BB	39	43100	53	43503	88	45488	1158	42747
71BT	45	60219	48	59842	124	60587	1527	56877
71TB	54	50365	63	50307	115	56224	1553	53598
71TT	56	48917	62	48573	125	48643	1341	47704
63BB	29	21944	35	21836	63	24303	593	23305
63BT	48	12492	47	12269	74	11898	539	11290
63TB	36	31272	49	32310	101	32581	1141	30302
63TT	40	48294	49	48131	99	48062	958	47017
62BB	28	15201	28	15117	52	16635	752	15743
62BT	36	31832	40	31136	85	31068	786	30387
62TB	35	51276	40	52372	76	52403	1118	49623
62TT	33	52869	35	52970	105	53032	1193	50695
61BB	50	27871	59	28271	122	28496	1309	26883
61BT	45	45349	49	45385	98	45491	909	43471
61TB	44	41001	55	41620	105	41972	1328	40261
61TT	33	58538	34	58518	94	58501	1290	55099
54BB	40	30055	46	29745	93	30515	1031	29583

54BT	48	57584	50	57460	89	57442	1012	52637
54TB	53	56007	58	55760	106	55918	1173	55568
54TT	57	59443	60	58352	129	58500	1683	55722
53BB	53	30498	56	29621	96	30818	888	29609
53BT	56	31670	59	28193	104	27996	918	30782
53TB	52	30259	58	30097	88	30295	1065	30056
53TT	59	53656	62	53733	122	54756	1549	54797
52BB	37	39161	42	38945	106	39288	1141	37367
52BT	53	61840	57	60896	112	61318	1398	58143
52TB	45	42113	49	42817	96	42792	1308	41162
52TT	49	40622	58	38010	121	40161	1179	39421
51BB	35	24268	34	23906	75	23903	692	22228
51BT	31	26197	32	26538	76	26485	772	25092
51TB	76	46329	86	45284	154	47511	1456	46179
51TT	55	63926	52	63536	124	63876	1489	61642
44BB	63	38679	73	38813	112	39371	1183	38009
44BT	58	37000	62	36988	106	36968	754	35174
44TB	70	52080	82	51534	150	52692	1487	53877
44TT	50	70683	58	70776	117	70933	976	67818
43BB	35	19832	40	19922	70	19916	545	18960
43BT	34	10374	36	10373	64	10363	350	10070
43TB	64	65379	72	62256	92	61641	1080	60458
43TT	37	27171	38	26337	51	25960	724	24362
42BB	51	58478	62	65854	105	65774	1092	62708
42BT	72	63479	80	63274	117	63565	927	60454
42TB	57	59708	66	58965	90	58954	767	57523
42TT	86	55749	94	55077	148	55241	946	53748
34BB	63	40183	66	35389	93	34747	845	42099
34BT	50	49492	55	49355	104	49303	800	46557
34TB	38	25344	40	25009	88	24820	888	23864
33BB	91	37574	102	37797	147	39258	1053	38339
33BT	59	37557	62	37499	106	37602	889	36959
32BB	76	34133	82	33602	140	34119	1088	33352

32BT	33	26628	34	27179	77	27141	552	25622
32TB	46	17896	54	18240	110	18258	732	16737
32TT	41	45271	40	45196	90	45120	862	42089
31BB	39	70703	42	70910	71	70593	660	66750
31BT	17	70476	21	70510	36	70474	532	66437
31TB	63	45362	74	44691	115	44548	877	45432
31TT	45	100183	46	100147	80	100121	966	94402
24BB	49	37489	54	37330	99	37318	942	35469
24BT	50	86654	55	86593	115	87601	1351	82942
24TB	56	51240	62	51940	102	51961	1243	50512
24TT	70	46195	76	46880	132	47690	1237	46108
23BB	42	33280	49	37252	86	37027	1023	36006
23BT	30	11495	31	11490	68	11556	531	10708
23TB	55	41801	60	41476	87	41243	1296	39787
23TT	50	45559	52	45557	96	45541	1054	43330
22BB	49	18082	54	18059	76	18398	582	18471
22BT	42	52360	45	52386	91	52369	1122	49715
22TB	35	25387	36	25387	56	25381	668	23759
22TT	36	39708	35	39707	77	40955	922	40176
21BB	25	39953	26	39092	48	39075	548	37262
21BT	58	58082	61	58141	112	58098	1163	55953
21TB	52	32874	54	32868	96	32891	1035	31675
21TT	47	72547	49	72390	83	72276	1279	70567
14TB	55	53928	57	53801	103	53791	1507	51466
14TT	65	52935	74	53189	133	53628	1256	52006
13BB	47	35082	50	35072	92	35043	1002	33794
13BT	61	44511	64	44498	123	44369	1072	42615
13TB	50	67943	57	67289	89	67083	1325	64498
13TT	59	44579	66	45138	113	45072	1201	44208
12BB	49	38156	57	39549	107	40232	1003	38576
12BT	40	63310	41	63273	91	63255	891	60010
12TB	48	65616	56	66348	112	66272	1871	63457
12TT	47	46700	47	46176	109	46182	1542	45554
