# Supplementary material for: A confidence interval analysis of sampling effort, sequencing depth, and taxonomic resolution of fungal community ecology in the era of high-throughput sequencing
Source: PLoS One. 2017 Dec 18;12(12):e0189796. doi: 10.1371/journal.pone.0189796 (PMC5734782; doi:10.1371/journal.pone.0189796)
Supplement: S3 Table — (PDF) [file pone.0189796.s012.pdf]

S3 Table. Number of operational taxonomic units (S obs) and sequence reads per sample (read number) at different ITS1 similarity cut-off thresholds for *P. torreyana* needle samples.

Site and tree #	S obs 90%	read number	S obs 95%	read number	S obs 97%	read number	S obs 99%	read number
Santa Rosa Island 1	27	36319	29	35730	34	34362	80	26008
Santa Rosa Island 3	27	22820	27	22559	29	21899	50	17361
Santa Rosa Island 4	34	59555	42	55876	46	53723	208	40019
Santa Rosa Island 5	24	30253	31	34245	30	52562	87	23366
Santa Rosa Island 6	33	35288	49	34592	55	33220	195	25288
Santa Rosa Island 7	30	11117	30	10968	37	10609	116	8077
Santa Rosa Island 8	50	42565	48	40926	57	38671	179	30625
Santa Rosa Island 9	39	63977	43	62476	47	60282	148	48981
Santa Rosa Island 10	31	32730	34	32063	41	30714	132	24540
Santa Rosa Island 11	29	18097	28	17686	32	17284	93	12283
Santa Rosa Island 12	29	9120	30	8990	30	8648	73	6451
Santa Rosa Island 13	26	8716	33	8543	34	8299	89	5771
Santa Rosa Island 14	28	29986	30	29551	32	28662	102	20958
Santa Rosa Island 15	23	27757	25	27519	29	26840	72	15400
Santa Rosa Island 16	30	56220	35	55041	35	53440	118	42517
Santa Rosa Island 17	36	97135	38	95982	39	93178	117	76923
Santa Rosa Island 18	25	54179	22	53768	24	52555	78	40057
Santa Rosa Island 19	30	89019	29	88035	31	85477	64	70132
Santa Rosa Island 20	14	28640	18	28270	22	27371	68	19992
Santa Rosa Island 21	37	16180	37	15916	39	15413	120	9538

Santa Rosa Island 22	26	45082	26	44475	29	42973	86	33028
Santa Rosa Island 23	24	38856	25	38269	27	36936	65	2849
Santa Rosa Island 24	17	49622	21	49049	18	48027	51	28891
Santa Rosa Island 25	18	32108	22	31816	20	30832	82	23954
San Diego 1	32	24803	33	23830	43	23350	123	17834
San Diego 2	31	30481	33	29927	37	28929	118	21822
San Diego 3	33	37331	39	36795	41	35637	130	25587
San Diego 4	19	11491	22	11376	20	11108	37	6691
San Diego 6	18	49996	19	48652	21	46042	31	36085
San Diego 7	13	66754	19	64748	22	60830	70	48968
San Diego 8	34	55602	44	54163	54	52230	186	36638
San Diego 9	26	41031	27	40338	38	38813	157	29259
San Diego 10	32	57333	34	56370	38	54593	155	42458
San Diego 11	19	57669	26	57049	27	55613	102	40047
San Diego 12	25	16035	27	15766	30	15294	102	12186
San Diego 13	28	44828	34	43569	36	41840	120	29629
San Diego 14	23	33256	30	32868	28	31937	75	23102
San Diego 15	25	24793	22	24370	29	23470	97	17112
